# Semi-Automated Quantification of Shape-Similar Features in Additively Manufactured Stainless Steel Components Using Immersion Ultrasound

**DOI:** 10.3390/ma19204281

**Published:** 2026-10-10

**Authors:** Savannah M. Rose, David A. Jack

**Affiliations:** 1 Materials Science and Engineering, Baylor University, Waco, TX 76798, USA; savannah_rose1@baylor.edu; 2 Mechanical Engineering, Baylor University, Waco, TX 76798, USA

**Keywords:** additive manufacturing, fused filament fabrication, feature quantification, image processing, closely packed feature identification

## Abstract

Additive manufacturing (AM) deposits material layer-by-layer, allowing for more geometric freedom than traditional manufacturing methods and reducing waste material. One barrier to industrially using AM components for structural applications is the ability to quantify defects and qualify components post-manufacture. This paper aims to demonstrate a feature identification and geometric quantification method from multiple ultrasonic waves captured in a raster pattern for AM components with multiple closely packed, similarly shaped features extruded on a component’s interior surface. This approach’s uniqueness is a means to quantify different feature dimensions, specifically the hexagonal longitudinal diagonal and extrusion height, within a dense field of features using a semi-automated image processing method. This approach is demonstrated on stainless steel components manufactured via the fused filament fabrication process utilizing a polymer carrier that is removed during post-processing. Results are validated by characterizing the as-manufactured geometric dimensions utilizing optical microscopy for features with nominal long diagonals of 4.06 mm and nominal extrusion heights ranging from 1 mm to 2 mm. The presented image processing technique has a planar measurement quantification error ranging from −0.1 mm to −0.7 mm and a feature extrusion height quantification error ranging from −0.30 mm to 1.24 mm across a range of sample thicknesses from 2 mm to 7 mm.

## 1. Introduction

Repeating features are common in various manufactured components. One such component type is honeycomb sandwich structures, where a hexagonal pattern occurs in lightweight components while maintaining high performance [1]. However, this method uses traditional manufacturing techniques. Of interest in the present study is a component fabricated utilizing additive manufacturing capabilities, specifically a component with internal channels such as in Ref. [2]. These repeating internal features, inaccessible to traditional inspection and qualification methods, are of interest, but current methods tend to identify only large defects relative to the feature dimensions [3]. Classical non-destructive evaluation (NDE) focuses on inspecting “indicators” or “defects”. However, internal features must be dimensionalized to determine whether they fall within a specific tolerance to perform correctly, especially in confined spaces such as cooling channels or other repeating geometric arrays. When there are large quantities of features, each feature needs to be assessed for dimensionality from within a group of hundreds or even thousands of features to determine feature accuracy.

Additive manufacturing (AM) is a collection of manufacturing processes by which components are created layer by layer [4]. In the present study, we focus on metal fused filament fabrication, also known as atomic diffusion additive manufacturing (ADAM). This is a novel process whereby the filament is a polymer matrix with a metal powder suspended within it [5,6]. This allows metal to be deposited in a lower-cost manufacturing process than other AM processes, such as powder bed fusion or directed energy deposition [7]. Once deposited, the component is post-processed in a chemical bath to remove the polymer and thermally sintered in an oven to combine the metal particles [8]. The result is a fully metallic component but is susceptible to warpage, delamination, and feature loss.

Nondestructive testing (NDT), also known as nondestructive evaluation (NDE), is a collection of methods for qualifying components for use, both after manufacturing and during service. Some important aspects in qualification are verifying designed dimensions and finding defects occurring during manufacturing. There are many types of NDT, such as X-ray computed tomography (CT), infrared thermography, flash thermography, eddy current, radiography, and ultrasound [9]. The method of interest in the present study is ultrasound testing (UT) due to its ease of use and lower cost relative to other NDT methods. It can obtain volumetric data, unlike eddy current inspection approaches for most metallic systems, and can be easily incorporated into a gantry or robotic system for automated inspection systems, unlike X-ray CT, which requires an enclosure.

Ultrasound testing (UT) utilizes ultrasonic sound waves from a piezoelectric element to send and receive signals, which, when interpreted, give insight into the internal structure of a component [10]. There are several common types of UT based on the sound wave generation method, such as single-element, phased array, and laser ultrasound, as well as different modalities for sound transmission such as contact, immersion, and air-coupled [11]. In the present study, we utilize a single-element, spherically focused transducer due to the ability to generate and capture high-fidelity waveforms and reflections off of small spatial features. Spherically focused transducers are typically operated in through transmission, pitch/catch, or pulse-echo mode [11]. Through transmission sends and receives signals using two transducers placed on opposite sides of a component, whereas pitch/catch uses two transducers on the same side of the component at different angles to send and receive shear waves. The through-transmission approach requires access to both sides of a surface of a part, which often is not feasible in the complex geometry components afforded by the additive manufacturing process. The pitch–catch approach is often limited to surface and near-surface indicators, and does not lend itself to feature geometry quantification of complex indicators. The pulse/echo approach utilizes a single transducer to both send and receive longitudinal acoustic waves. The advantage, for the present study, is that pulse/echo only requires access to a single side of the component [10] while also allowing for a depth quantification of an indicator.

Image processing has been performed for additive manufacturing components in situ with camera images on individual printed layers [12]. Saini et al. [12] used optical images and image processing methods to detect individual features layer by layer in situ in the fused filament fabrication (FFF) process for different shapes. These techniques included simple thresholding, adaptive thresholding, Sobel edge detection, Canny edge detection, and the watershed transform. They used these image processing techniques on isolated circular, rectangular, and triangular cavities in subsequent layers in an FFF print to determine if the features could be detected. They determined that simple thresholding and adaptive thresholding had the best performance among the techniques investigated, followed by Canny edge detection, the watershed transform, and Sobel edge detection. For low-contrast features, they found that the adaptive thresholding and Sobel edge detection methods had reduced performance compared to the high-contrast features. However, simple thresholding and the Canny edge detection methods were able to detect the external sample edges.

Phased array ultrasound has also been used to detect different shapes of features at various depths within metal powder bed fusion components [13]. Honarvar et al. [13] used phased array ultrasound to inspect multiple shapes of cavity features embedded in laser powder bed fusion metallic components. Such features were rectangular, circular, and hexagonal in the plane of inspection. They found that features were detectable down to 0.75 mm. They measured all features they detected and compared them to measurements taken from X-ray computed tomography; however, all ultrasound measurements were manual measurements. Our study differs as the features are extruded features rather than cavity features, and the measurements are automated measurements using image processing techniques.

Image processing techniques have commonly been used with optical images to detect defects in powder bed fusion components [14,15]. Abdelrahman et al. [14] used image segmentation and registration to determine defects in situ for powder bed fusion components using camera images. They used image registration and segmentation based on slices from a 3D CAD model of the printed component. They used the level sets method for segmentation and defect quantification during the printing process. They found the sensitivity of the system to be 0.915 and the specificity of the system to be 0.840. The system sensitivity is a quantitative measure of how well the method determines if a section contains a potential defect relative to another section within the same layer, whereas the system specificity relates to how well different layers are correctly identified as not having defects. Lu et al. [15] used an in situ image processing algorithm to determine the porosity in cylinders manufactured with selective laser melting (SLM). The algorithm was used after each layer was printed. The algorithm consisted of generating a binary mask, averaging the grayscale of the cylinder in the mask region on the original image, finding the difference between the mask and the grayscale average, adaptive histogram equalization, and thresholding for binarization.

One common feature detection technique for ultrasound testing is processing the C-scan data [16,17]. Nargis et al. [16] used immersion ultrasound to automatically quantify FOD area in carbon fiber laminates. They quantified the FOD area using image processing techniques on a C-scan. Specifically, they applied a filter to the C-scan, binarized the data using a threshold, closed the mask, and found the edges of the closed area. Similarly, Ravindranath et al. [17] used immersion ultrasound to inspect barely visible impact damage (BVID) in composite laminates using image processing techniques. They found the gradient of the C-scan, binarized the C-scan using a defined threshold, removed small surfaces, closed the surfaces, and extracted the edges of the damage throughout the laminate thickness.

Ultrasound can be used to inspect features designed into components. Taherkhani et al. [18] used phased array ultrasound to detect and quantify QR codes that were made via cavities left inside metal PBF components that were filled with unmelted powder. They performed this study on samples on “up skin” and “down skin” samples, where the surface roughness changed depending on which surface it was being inspected from. They combined three to six consecutive layers together from the UT data to create an image and perform image processing on this composite image. First, they crop and rotate the image to obtain the correct size and orientation of the image. They also perform a “color transformation” to make the originally white pixels black and vice versa. Then, they binarize the image and apply a median filter to smooth the image. Then, the histogram of gradients (HOG) method and transfer learning method are used to detect the QR code pattern. Our study differs in that, although both look at internal features that are inaccessible to traditional methods, they look at cavity features rather than extruded features. Also, this study requires using several layers to create a composite image, whereas this study uses a single slice in time to detect features.

The approach in the present manuscript for feature identification and quantification is unique because it does not rely on calibration blocks with features of specific geometries and sizes to quantify features. Previous efforts identify a single indicator in a region without other indicators, whereas the present study seeks to quantify indicators in a large field of closely packed indicators, each of which may be of a similar size. In addition, previous research has focused on flat bottom hole defects and other inset features inherent to or well defined for subtractive manufacturing, such as Ref. [19] who utilized phased array to study rough surfaces and the sensitivity of detecting 2 and 5 mm through holes. Similarly, ref. [20] investigated the use of laser ultrasound to detect through holes between 0.4 mm and 2 mm in a powder bed fabricated specimen. Similarly, Ref. [21] inspected components fabricated through the ADAM process utilizing an immersion ultrasound, and artificially introduced defects using a drilling process. In each case, a solitary defect was placed in a larger region without a defect, and the objective was to detect the inserted defect. Conversely, the present manuscript focuses on interior extrusions, adding both manufacturing complexity and an inspection challenge due to the increased thickness of the feature. This is further complicated as the acoustic waveforms off the flat-bottom hole are from a flat and highly reflective surface, but with an extrusion there is significantly less signal contrast.

The current paper uses an image processing approach for feature identification within the captured data sets. This avoids the need for machine learning, which requires significant data for training of each individual feature size and type, along with new training for each material type, whereas the presented method is agnostic to feature size and type in that it does not require a priori knowledge of the feature shape and size, with only a minimal change in the signal threshold value based on material types. The approach is demonstrated on stainless steel specimens additively manufactured using the fused filament fabrication process, which requires a post-fabrication sintering process whereby the polymer binder is removed, and the steel powder is consolidated.

## 2. Materials and Methods

### 2.1. Sample Manufacturing

**Figure 1:**
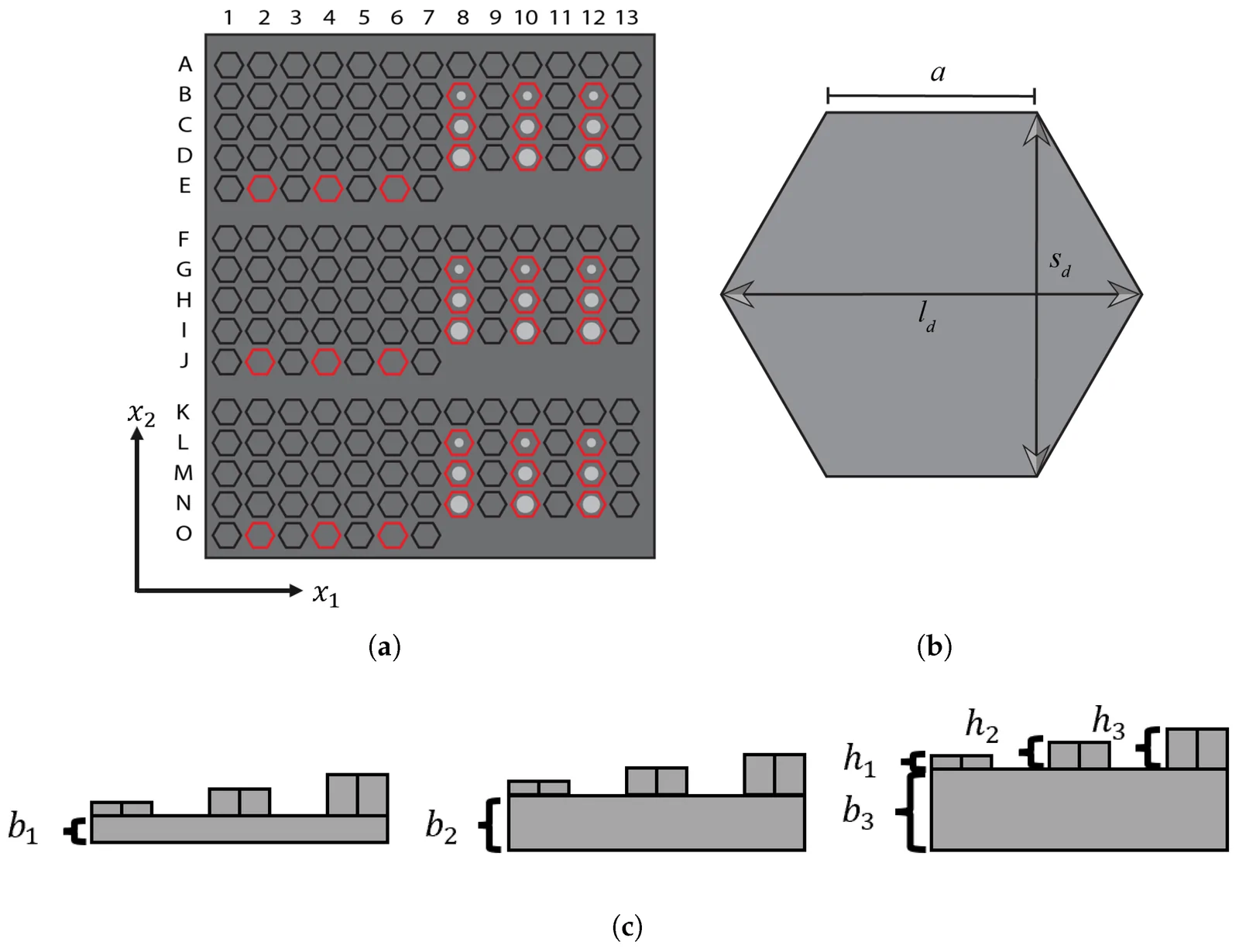
Schematic showing the sample features, including the hexagon sets: (**a**) top-down view (note that the hexagons with the red border are not used in the present study), (**b**) hexagon dimension notations, and (**c**) side view of the hexagon set extrusion. Note that regions with a red border are not used in the present study, as they have intentional defects.

A set of samples for inspection was designed around a component with multiple repeating extrusions on the back side of an additively manufactured structure. The concept is that a “well-made” component would contain a series of internal extruded hexagons in some repeating pattern, each with a separation less than the major axis of the hexagon. Each sample, such as that shown in Figure 1, is composed of a base upon which three sets, or clusters, of hexagons were extruded. Three samples were manufactured, each with a different nominal base thickness, where $b_{1}=1.27$ mm, $b_{2}=2.54$ mm, and $b_{3}=4.83$ mm. Each cluster of hexagons on a single sample was extruded to a different nominal height: $h_{1}=1.02$ mm, $h_{2}=1.52$ mm, and $h_{3}=2.03$ mm. The long diagonal of the hexagonal features, $l_{d}$, is 4.06 mm, and the gap spacing between the points of neighboring hexagons is 0.51 mm. The sample outer dimensions are 61 mm × 66 mm. The hexagon short diagonal is defined as $s_{d}$, and the hexagon side is defined as *a*, as seen in Figure 1b. The feature dimensions are summarized in Table 1. Notice that there are 36 hexagons with a red border in Figure 1. These hexagons have various indicators purposefully embedded within them and are not used in the present study. In total, there are 177 hexagonal features extruded on each sample, 59 in each cluster, of which 47 are used in the present study.

The samples were manufactured using the fused filament fabrication (FFF) approach, with manufacturing performed on an Ultimaker S5 (UltiMaker, Utrecht, The Neatherlands). A 2.85 mm diameter 17-4PH stainless steel filament from BASF (Ludwigshafen, Germany) was used that is 90% by weight stainless steel powder suspended in a polymer matrix [22]. The 17-4PH stainless steel is a precipitation-hardened martensitic stainless steel commonly used due to its high-temperature capabilities and high corrosion resistance in the aerospace and chemical industries [23,24,25]. The print profile recommended by Ultimaker was used for printing, specifically the recommended infill percentage of 105% and a layer height of 0.15 mm. A thin layer of adhesive is placed on a glass build plate prior to printing to ensure sufficient build plate adhesion and mitigate warping during printing. Following manufacturer specifications, debinding is performed in $HNO_{3}$ at 120 °C to remove the polymer binder [22]. Sintering follows in three steps: (1) heating to 600 °C at 5 K/min and holding for 1 h, (2) heating from 600 °C to 1300 °C at 5 K/min and holding for 3 h, and (3) cooling the furnace naturally in an atmosphere of either hydrogen or argon, according to the material manufacturer’s specifications [22]. This results in a densified metal component with minimal void content. During post-processing, the components shrink up to 20% in the plane of the print bed directions ($x_{1}$ and $x_{2}$ in Figure 1) and up to 24% in the through-thickness direction, according to the material manufacturer [22], which was taken into account for the present study. The samples used in this study were printed such that the flat base was printed on the build plate, and the hexagons were the last section printed. Post-processing can lead to significant part warpage of flat components due to internal stress being induced [8]. Warpage is exacerbated with thinner samples. Because of this, all samples were printed with a nominal base of 4.83 mm to minimize this warping. Then, to obtain the designed thickness, the base was post-processed by milling to 1.27 mm for the thin samples and 2.54 mm for the medium-thickness samples using a manual Grizzly (Bellingham, WA, USA) knee mill. It was observed from initial nondestructive inspections that the thickest base sample had a delamination on the bottom of the base from printing and post-processing; thus, it too was milled to remove the defect, with a final thickness of 4.80 mm.

### 2.2. Feature of Interest Measurement

#### Microscope Measurement

**Figure 2:**
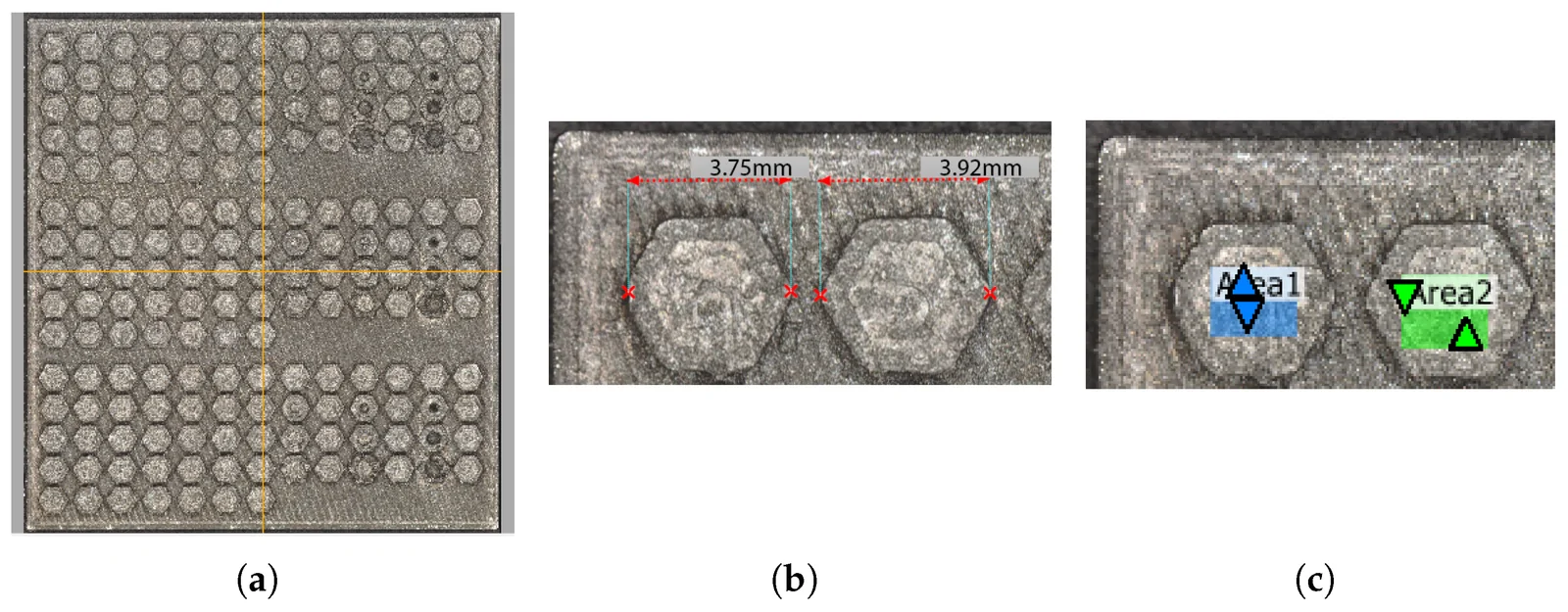
Optical microscope measurement method where (**a**) shows the reference plane selection method, (**b**) shows an example long diagonal measurement, and (**c**) shows an example region for thickness measurement relative to the sample base plate.

A VR3000 made by Keyence (Osaka, Japan) was used to measure the manufactured dimensions of the designed features in each sample. Two dimensions were measured: the long diagonal of the hexagon, $l_{d}$, and the extrusion height of the hexagon relative to the base plate, $h_{i}$, where i=1,2,3, as seen in Figure 1. Before taking any measurements, a reference plane was created on the sample by setting a surface profile such that the plane was parallel to the yellow lines placed along both the row and column of the hexagons, as shown in Figure 2a. Next, the Keyence measurements were verified by measuring with a NIST-traceable Geller micrometer, where the uncertainty in the measurements is based on 5 measurements taken with the Geller micrometer, based on ASTM E766 [26]. The expanded combined uncertainty, *U*, is calculated based on the uncertainty in the Geller micrometer, $U_{gellar}$, the uncertainty in the microscope, $U_{Keyence}$, the uncertainty in the individual measurements taken, $U_{measurements}$, and the coverage factor, *k* as

(1) $$U=k(U_{gellar}+U_{Keyence}+U_{measurement})$$

where the coverage factor was chosen as 3 to give a confidence level greater than 99%. The uncertainty for the measurements is calculated as

(2) $$U_{measurement}=\sqrt{\frac{\Sigma {\left(q_{i}-\bar{q}\right)}^{2}}{\left(n-1\right)}}$$

where $q_{i}$ is the value of each measurement taken, $\bar{q}$ is the average of all measurements taken, and n is the total number of measurements taken, which in this case was 5. From this, the expanded uncertainty in the direction of the long axis of the hexagons was determined to be 1.85 μm.

The long diagonal width of the hexagon from the microscope image was measured by manually selecting two points at each hexagon shown in Figure 2b. Next, a region in the center of the hexagon, such as shown in Figure 2c, was selected to measure the average height of the hexagon relative to the reference plane. This is then added to the base thickness, which is taken as the average base thickness from caliper measurements of three locations on the base. The arrows in Figure 2c show the maximum (as seen by the arrow pointing up) and minimum (as seen by the arrow pointing down) height locations within the selected region.

### 2.3. Ultrasound Inspections

**Figure 3:**
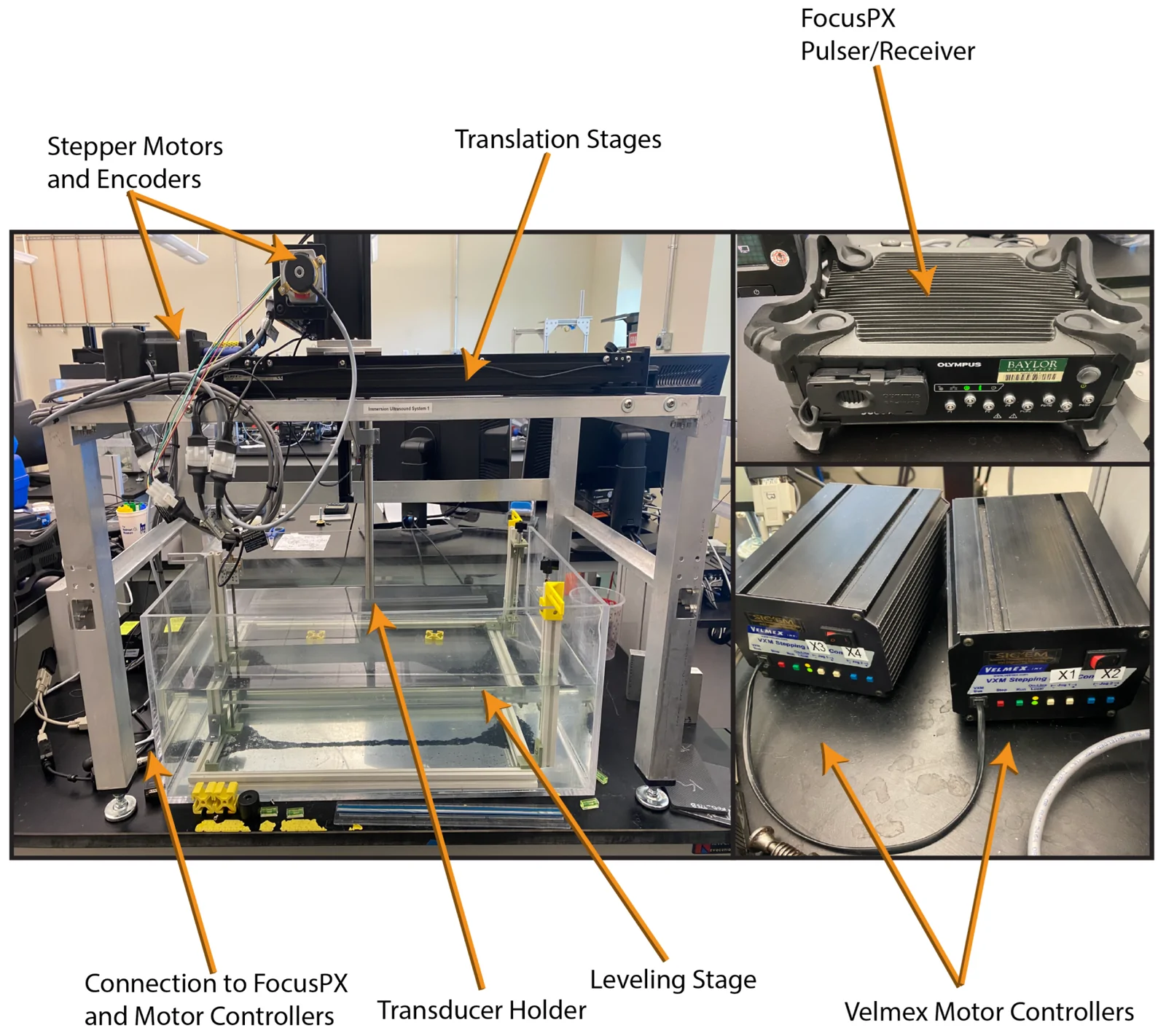
Ultrasound immersion system showing the immersion tank, FocusPX pulser/receiver, and Velmex controllers.

Ultrasound inspections of the samples were performed on the custom immersion ultrasound system shown in Figure 3. This system has two orthogonal translation stages to move the transducer across the component surface, with a third orthogonal axis to adjust the transducer separation relative to the part to set the focal plane. The motors are controlled using two connected motor controllers (Velmex, Bloomfield, NY, USA). A FocusPX pulser/receiver (EVIDENT, Waltham, MA, USA) was used to excite the transducer and receive the reflected signals without applying filtering to the signal. All inspections were performed in pulse/echo mode, such that a single transducer sends and receives the signals with a digitization frequency of 100 MHz, with a resolution of 0.2 mm in both the scan, $x_{1}$, and the index, $x_{2}$, directions.

**Figure 4:**
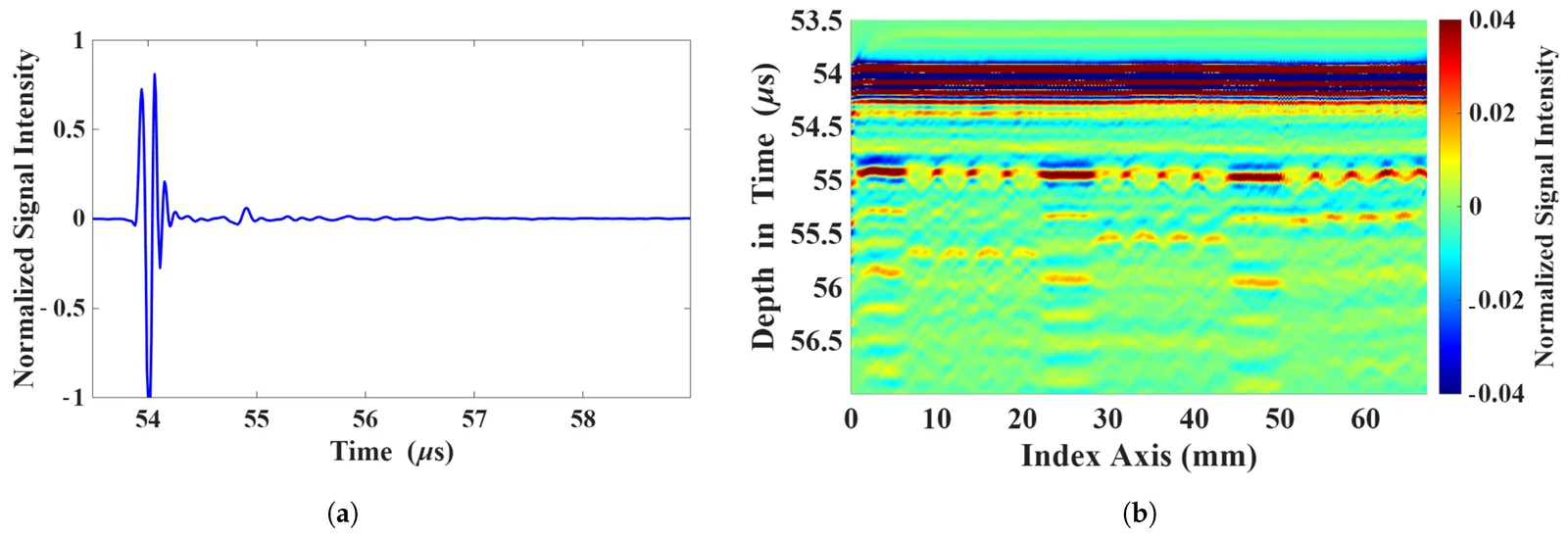
Example A-scan and B-scan: (**a**) raw A-scan with the front wall at 54 μs and (**b**) B-scan with a side view of the hexagon feature steps.

At each raster location, the voltage measured by the transducer from the reflection wave is recorded. This process is repeated sixteen times and averaged at each raster location to remove random electrical noise, the result of which is termed an A-scan. Figure 4a shows such a representative normalized A-scan, where the initial reflection of the front wall is seen near 54 μs, and the reflection of the back wall is seen shortly afterward, around 55 μs. The A-scan is normalized against the largest voltage magnitude from all captured A-scans of a given sample. A series of A-scans along a single projection is termed a B-scan, such as that shown in Figure 4b of a representative projection along the index axis, $x_{2}$, along the 11th column of features from Figure 1 for the medium base sample. Notice in Figure 4b that the time t=54μs corresponds to the initial reflection wave of the front wall. As can be seen, the large interval between the groups can be observed for $x_{2}∼5$ mm, $x_{2}∼25$ mm and $x_{2}∼50$ mm around 55 μs. Similarly, between the spans of the clusters, the individual hexagonal short diagonals, $s_{d}$, can be seen, where there are 4 hexagons observed within each cluster in the 11th column of the sample. The B-scan shown in Figure 4b has been scaled in intensity from a range normalized to ±1 to an amplified range of [−0.04,0.04] to highlight the back-wall reflections of the hexagonal features of the samples of interest in this study that appear slightly before 55 μs. Note that typically a B-scan is along a single index location and a D-scan is along a single scan location; however, in this case, the B-scan is denoted as being along the scan axis. In Section 4, a third type of visualization of the acoustic data will be shown, termed a C-scan, essentially a top-down view of the data to provide a sense of the features at a given depth into the sample.

#### Ultrasound Parameter Selection

The selected transducer for the present study is a PTF (Evident, Waltham, MA, USA), spherically focused 10 MHz immersion transducer with a 38.1 mm focal length and 12.7 mm active element diameter. The excitation voltage was 190 V for all inspections, and the pulse width was determined based on $p=\frac{1}{2f}$, where *p* is the pulse width in seconds and *f* is the peak transducer frequency in inverse seconds. All inspections were performed in a laboratory environment where the temperature ranged from 68 °C to 72 °C. The samples were inspected such that the hexagonal features were on the opposing side to that of the transducer to satisfy the scenario that the hexagonal features would be on a surface that could not be easily accessed for an inspection. An area larger than the sample was included in the scan region such that the transducer traversed entirely off the component; afterward, the data were trimmed to only include the sample. This ensured that the entire sample was inspected. For the present study, the transducer was focused on the front wall of the component to align with the scenario that the part thickness was not known a priori. This resulted in blurring of the resulting C-scans. Further advances can be made in the future if one were to relax the assumption that there is no knowledge of the expected part thickness.

### 2.4. Feature Quantification Approach

The raw signal intensity captured from the transducer is denoted as $I(t,x_{1},x_{2})$, where *t* is the signal time, $x_{1}$ is the scan axis in space across the sample, and $x_{2}$ is the index axis in space across the part. Before analysis, the captured data is trimmed spatially to remove points in $\left(x_{1},x_{2}\right)$ that are off the sample. Next, the front wall time, $\hat{t}\left(x_{1},x_{2}\right)$, is found by finding the first intensity value that is greater than a predefined threshold, β, as

(3) $$\hat{t}\left(x_{1},x_{2}\right)=\mathrm{first}\left\{t:|I(t,x_{1},x_{2})|>\beta \right\}$$

For this study, a value of β=0.5 is used, as the typical front wall signal intensity was nominally 80–85% of saturation and the noise floor was less than 1%. As such, any value between β=0.1 and β=0.6 has a nominal change in the results.

Next, a two-dimensional polynomial $f(x_{1},x_{2})$ is fit to the detected front surface $\hat{t}\left(x_{1},x_{2}\right)$ to correct for any minor warpage of the part during manufacturing and to correct for any small bubbles that are present on the surface. In this study, a third-order polynomial was found to be sufficient for the thin and medium samples with an $R^{2}∼0.999$, while a zeroth-order polynomial was sufficient for the thick base sample as no significant warpage was observed. The adjusted time for the front wall reflection is then defined as

(4) $$\bar{t}\left(x_{1},x_{2}\right)=t\left(x_{1},x_{2}\right)-\left\{\begin{array}{cc} \hat{t}\left(x_{1},x_{2}\right), & \mathrm{if}\;|\hat{t}\left(x_{1},x_{2}\right)-f\left(x_{1},x_{2}\right)|\;<\;\alpha \\ f(x_{1},x_{2}) & \mathrm{else} \end{array}\right.$$

where subtracting the second half of the equation from $t(x_{1},x_{2})$ shifts the onset of the rise of the A-scan signal to the front wall. In Equation (4), the value of α=0.1 μs was found to be acceptable, as it removed any outliers caused by scratches, porosity, or microbubbles on the surface.

Next, we seek to define the base of the extrusion plane spatially, but only in regions where there is no extrusion. To perform this, we first capture the intensity of the back wall reflection wave $\bar{I}\left(x_{1},x_{2}\right)$, which is defined as

(5) $$\bar{I}\left(x_{1},x_{2}\right)=\max_{\bar{t}\geq \Delta t}\left\{\left|I\left(\bar{t},x_{1},x_{2}\right)\right|\right\}$$

along with the corresponding time of the back wall reflection wave

(6) $$\tau \left(x_{1},x_{2}\right)=\left\{\bar{t}\left(x_{1},x_{2}\right):\max_{\bar{t}\geq \Delta t}\left\{\left|I\left(\bar{t},x_{1},x_{2}\right)\right|\right\}\right\}$$

where Δt in Equations (5) and (6) is selected sufficiently large as to avoid any signal from the front wall echoes, but not so large as to pass the onset of the base wall echo. In the present study, a value of Δt=0.5µs was selected. Next, the base of the extrusion is identified by gating the back wall of the reflection wave as

(7) $$\bar{\tau }\left(x_{1},x_{2}\right)=\left\{\begin{array}{cc} \tau (x_{1},x_{2}), & \mathrm{if}\;\gamma_{L}\leq \bar{I}\left(x_{1},x_{2}\right)\leq \gamma_{U}\\ \mathrm{NaN} & \mathrm{else} \end{array}\right.$$

where $\gamma_{L}$ and $\gamma_{U}$ are lower and upper threshold values prescribed by the operator for the base of the extrusion plane reflection wave intensity. The pair {$\gamma_{U},\;gamma_{L}\}$ is identified from the B-scan, such as shown in Figure 4b. Observe in the figure that the base of the extrusions is around 55 μs, where the typical intensity of the base of an echo off the base of the extrusions was found to range from a lower intensity of $\gamma_{L}=0.04$ to an upper intensity of $\gamma_{U}=0.06$. For the thin sample, the intensity of the echo off the base of the extrusions was found to range over {$\gamma_{U},\gamma_{L}$} = {0.02,0.05}, whereas for the thick sample the pair {$\gamma_{U},\gamma_{L}$} = {0.02,0.027} was used.

**Figure 5:**
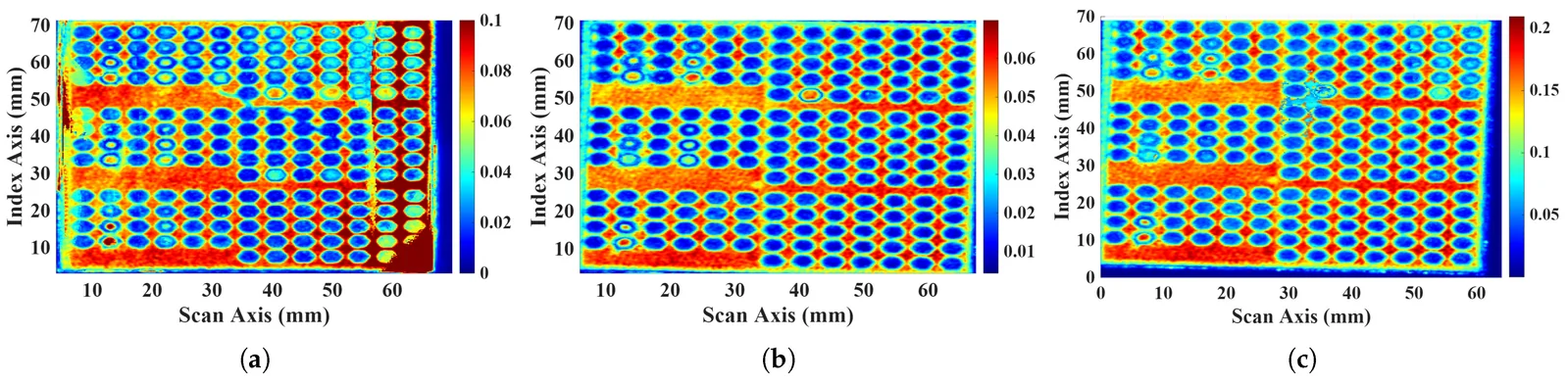
Raw C-scans at the base for (**a**) the thinnest base sample, (**b**) the medium base sample, and (**c**) the thickest base sample.

Next, a polynomial $g(x_{1},x_{2})$, selected to be the same order as the front wall polynomial $f(x_{1},x_{2})$, is fit to the base of the extrusion time $\tau (x_{1},x_{2})$. Finally, the C-scan, a planar visualization of the raster data at a given depth, is defined as

(8) $$c\left(x_{1},x_{2}\right)=\max_{\bar{t}\left(x_{1},x_{2}\right)\in \left\{\tilde{\tau }\left(x_{1},x_{2}\right)-\tau_{1},\tilde{\tau }\left(x_{1},x_{2}\right)+\tau_{2}\right\}}\left\{\left|I\left(\bar{t},x_{1},x_{2}\right)\right|\right\}$$

where $\Delta \tau =\tau_{2}-\tau_{1}$ is termed the gate and, in the present study, a value of 0.4 s was used; $\tau_{1}$ is the offset time used for the gate before the back wall, which in this study was 0.25 μs. The value $c(x_{1},x_{2})$ is, in essence, the maximum magnitude signal intensity of the acoustic wave on the plane that contains the base of the extrusions. This C-scan at the base of the extrusion plane for each sample is shown in Figure 5, where the color bar shows the maximum intensity amplitude within the gate of the A-scan at each point. Higher intensity values show where a reflection occurred due to an acoustic impedance difference at a material interface, such as between steel and water. The low intensity values occur within the hexagonal features because the sound wave is still traveling through the same material, in essence deeper into the part, and no reflected wave is observed. Since all hexagons begin extruding at the same depth, they are all visible at the base location. In essence, it is the lack of an echo that is the indicator of the presence of a hexagon extrusion. Note that several of the printed holes are visible at the extrusion base, as they were designed to penetrate into the base plate. The thinnest sample shows sharper contrast at the edges of the hexagons than the medium base thickness sample, which has sharper contrast at the edges than the thickest base sample. This is due to less attenuation and the fact that the focal plane is the top surface of the part and not the extrusion base.

Once the C-scan is generated, a rigid body rotation and translation are performed such that the origin is on the bottom-left corner of the sample and the repeating hexagons align along the coordinate axes. The coordinate transformation is given by $\langle {\bar{x}}_{1},{\bar{x}}_{2}\rangle =\underline{\underline{R}}\langle x_{1},x_{2}\rangle +\underline{T}$, where $\underline{\underline{R}}$ is the standard rotation tensor and $\underline{T}$ is a rigid body translation. The only sample without a transformation was the thin sample.

**Figure 6:**
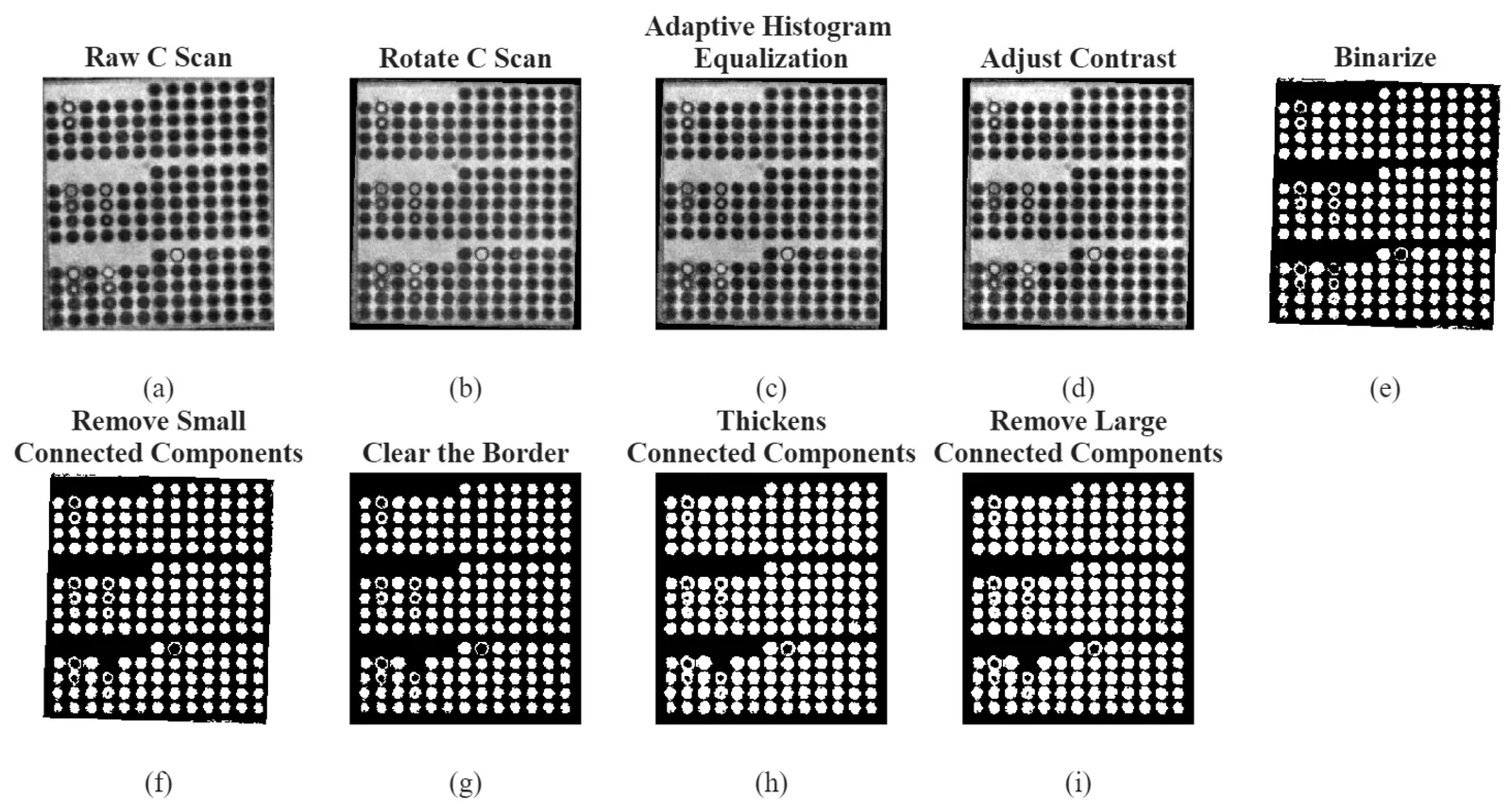
Example images showing the algorithm steps where (**a**) is the raw C-scan, (**b**) is the rotated C-scan, (**c**) is the adaptive histogram equalization step, (**d**) is the image contrast adjustment step, (**e**) is the binarization step, (**f**) is the remove small components step, (**g**) is the clearing borders step, (**h**) is the thickening step and (**i**) is the remove large components step.

Then, several image processing techniques are applied to the C-scan to separate out the hexagonal features. The first step to the approach used was adaptive histogram equalization using the MATLAB (R2025a) function adapthisteq, which performs contrast-limited adaptive histogram equalization (CLAHE) based on Zuiderveld’s work [27]. The C-scan from the adaptive histogram equalization $c_{H}\left({\bar{x}}_{1},{\bar{x}}_{2}\right)$ is shown in Figure 6c for the medium base sample.

Then, image contrast adjustment to remove statistical outliers is applied, creating $c_{I}\left({\bar{x}}_{1},{\bar{x}}_{2}\right)$, where the bottom 1% of values are set to 0, the top 1% of values are set to 1, and the values between the top and bottom 1% are linearly scaled. From Figure 6c,d, it can be seen that there is a sharpening of the edges around the darker hexagonal features and the brighter features within the darker features.

The next technique applied is binarization to form the binarized C-scan $c_{B}\left({\bar{x}}_{1},{\bar{x}}_{2}\right)$, defined as

(9) $$c_{B}\left({\bar{x}}_{1},{\bar{x}}_{2}\right)=\left\{\begin{array}{cc} 0, & \mathrm{if}\;c_{I}\left({\bar{x}}_{1},{\bar{x}}_{2}\right)>\delta \\ 1, & \mathrm{if}\;c_{I}\left({\bar{x}}_{1},{\bar{x}}_{2}\right)<\delta \end{array}\right.$$

and is shown in Figure 6e for the medium base sample. Standard binarization sets a value equal to 1 if the original value is greater than the defined threshold value and equal to 0 if the original value is less than the defined threshold value. However, as seen in Equation (9), the inverse of the standard binarization for an image is used because, when the hexagonal features are visible at the base of the sample, they are visible because there is an acoustic impedance difference caused by a material difference between the base and the water, so no reflection is seen at the hexagonal features at this point, but there is a reflection where there is no hexagonal feature. Once binarized, the data set is made of connected components of pixels. In this study, a δ value of 0.99 is used for all three samples. When changing the value from 0.99 to 0.95, there is a negligible change in the long diagonal measurement of less than 5%.

Next is the removal of noise and edge effects that are not the hexagonal features in the range of interest. In the present study, the nominal long diagonal was 4 mm; thus, we excluded any features with an equivalent long diagonal outside of the expected range. We chose the bounds to be rather large to demonstrate the robustness of the approach, and for the current study we discarded features with an equivalent long diagonal less than 1.75 mm and greater than 6.75 mm. An example of this is shown in Figure 6f. To remove any edge effects, any connected components occurring on the border of the data set are also removed, with an example shown in Figure 6g for the medium base sample. Then, thickening occurs to each connected component. Thickening is an operation where pixels are added to the outer edges of each connected component until neighboring components would be eight-connected [28]. This is shown as A⊙B=A∪(A⊛B) [29], where *A* is the data set being processed and *B* is a structuring element. In this case, A would be each connected component in the binarized C-scan, $c_{B}\left({\bar{x}}_{1},{\bar{x}}_{2}\right)$ following the border-clearing step, and *B* is a binary disk of radius 1. The result of this operation is shown in Figure 6h.

Following the image processing of the C-scan, the individual connected components in the binarized data set are measured. A bounding box is created around each connected component, and the long diagonal is cataloged.

Next, we desire to obtain the extrusion thickness of each individual hexagon. First, the time at which the back wall echo is needed. As the signal-to-noise ratio near the back wall is quite poor due to the scatter within the additively manufactured component, care must be taken to define the threshold value for the back wall. A threshold value ϵ is found by averaging the noise well after the last wave as

(10) $$\epsilon_{n}=\frac{\sum_{i=s}^{s+m}\sum_{j=1}^{N_{index}}\sum_{k=1}^{N_{scan}}\left|I\left({\bar{t}}_{i},{\bar{x}}_{1,j},{\bar{x}}_{2,k}\right)\right|}{mjk}$$

where ${\bar{t}}_{s}$ is the time to start averaging values for the noise floor in each A-scan, and *m* is the number of points to include in the average for the noise estimation. The noise floor is then scaled by a value μ to obtain a back wall threshold value η, such that η=μϵ. This back wall threshold value is used to find the hexagon height by finding the last value within an individual A-scan greater than the back wall threshold value. Values for μ of 5, 7, and 10 were investigated for the thick base sample. A value of 10 was not selected as it resulted in a higher threshold value than the back wall reflection for the medium cluster and the thickest cluster of hexagons. There was a nominal difference between the values of μ=5 and μ=7, so the value of μ=7 was arbitrarily selected and used for all samples. The back wall time at every spatial location is then obtained from

(11) $$\tilde{\tau }\left(x_{1},x_{2}\right)=\mathrm{last}(\bar{t}:|I\left(\bar{t},{\bar{x}}_{1},{\bar{x}}_{2}\right)\left|>\eta \right)$$

Next, the average back wall time within the $n^{\mathrm{th}}$ hexagon, termed ${\tilde{\tau }}_{n}$, is recorded as the average of the back wall times from the 9×9 grid centered about the centroid of the $n^{\mathrm{th}}$ hexagon. To calculate the thickness of the $n^{\mathrm{th}}$ hexagon, the time of the base wall $\bar{\tau }\left(x_{1},x_{2}\right)$ is subtracted from the time of the back wall of the hexagon ${\tilde{\tau }}_{n}$, resulting in the time of flight within the hexagon, from which the speed of sound of the metal, c= is used to convert time to a height of the $n^{\mathrm{th}}$ hexagon, $h_{n}$. The speed of sound value used in the present study was 4873 m/s. This was found by measuring the speed of sound of the material using the medium base sample at the base location where no hexagonal features are located, with an Olympus Epoch 650 and a 5 MHz contact transducer with a delay line.

## 3. Results and Discussion

To quantify the error between the optical measurements and the ultrasound (UT) measurements of individual features, the following simple definition is used:

(12) $$e_{l_{d}}\equiv l_{d_{UT}}-l_{d_{optical}}$$

where $e_{l_{d}}$ is the long diagonal error, $l_{d_{optical}}$ is the actual long diagonal measured from optical microscopy, and $l_{d_{UT}}$ is the long diagonal measured using the ultrasound method. The absolute value of the error is not used in the present study to highlight whether the value for the hexagon long diagonal width $l_{d_{UT}}$ is typically less than or greater than the actual hexagon long diagonal width. The following section presents the detection rate and the dimensioning of the extrusions as a function of the base thickness and extrusion height, or hexagon height, *h*.

The first sample presented is the medium thickness base sample that was detailed in Section 3 with the imaging results shown in Figure 6. For this sample, the presence of each of the hexagons used in the current study was detected correctly. The average error of the hexagon long diagonal width, $e_{l_{d1}}$, for the extruded hexagons in the thin region of the medium-thickness sample is −0.4 mm, whereas the average error is −0.3 mm and −0.5 for the medium and thick regions, respectively ($e_{l_{d2}}$ and $e_{l_{d3}}$). The average error across the entire sample, $e_{l_{d}}$, is −0.4 mm.

**Figure 7:**
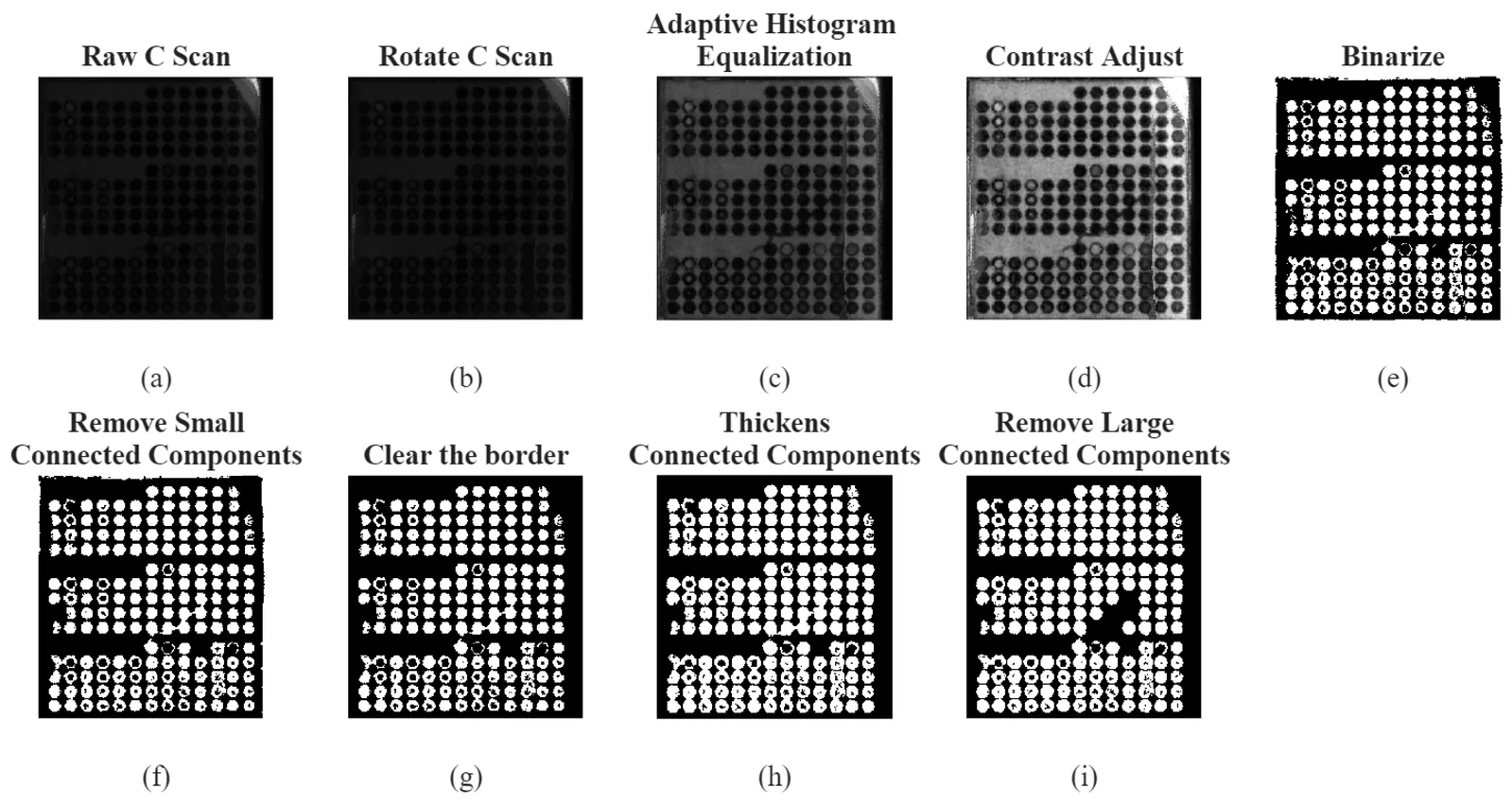
Thin sample image processing steps for feature detection.

The thin base sample feature detection results for the image processing visualization are shown in Figure 7. In the thick region of the sample, 45 of 47 features were detected correctly with an average error, $e_{l_{d3}}$, of −0.3 mm. The medium region of the thin base sample has several irregularities that were not anticipated, specifically the presence of the crack in the sample. This crack had the net effect of gathering multiple features into one large collective feature, which was removed from calculations as it was larger than the greatest allowed feature. Although it is helpful that our algorithm identified the features with a crack, those are removed from the current study’s assessment. With those features removed from consideration, the algorithm successfully identified and measured all but 1 of the hexagons in the medium extrudate region of the thin sample. The crack in the medium extrudate region can be observed in Figure 7 after contrast adjustments are made and is most noticeable in Figure 7h. The error, $e_{l_{d2}}$, for the medium extrudate region is −0.3 mm. Lastly, the thin region of the thin base sample has several pairs of hexagons that are grouped into one feature. They remain as captured features; however, the pixel count is less than the cutoff threshold discussed in Section 3. These values were removed from measurement for the long diagonal width measurement comparison. The thin extrudate section had 41 of 47 features detected; 6 of these 41 were paired features. When removing the incorrectly detected and paired features, the thin extrudate region had an average error, $e_{l_{d1}}$, in the feature long diagonal width of −0.1 mm.

**Figure 8:**
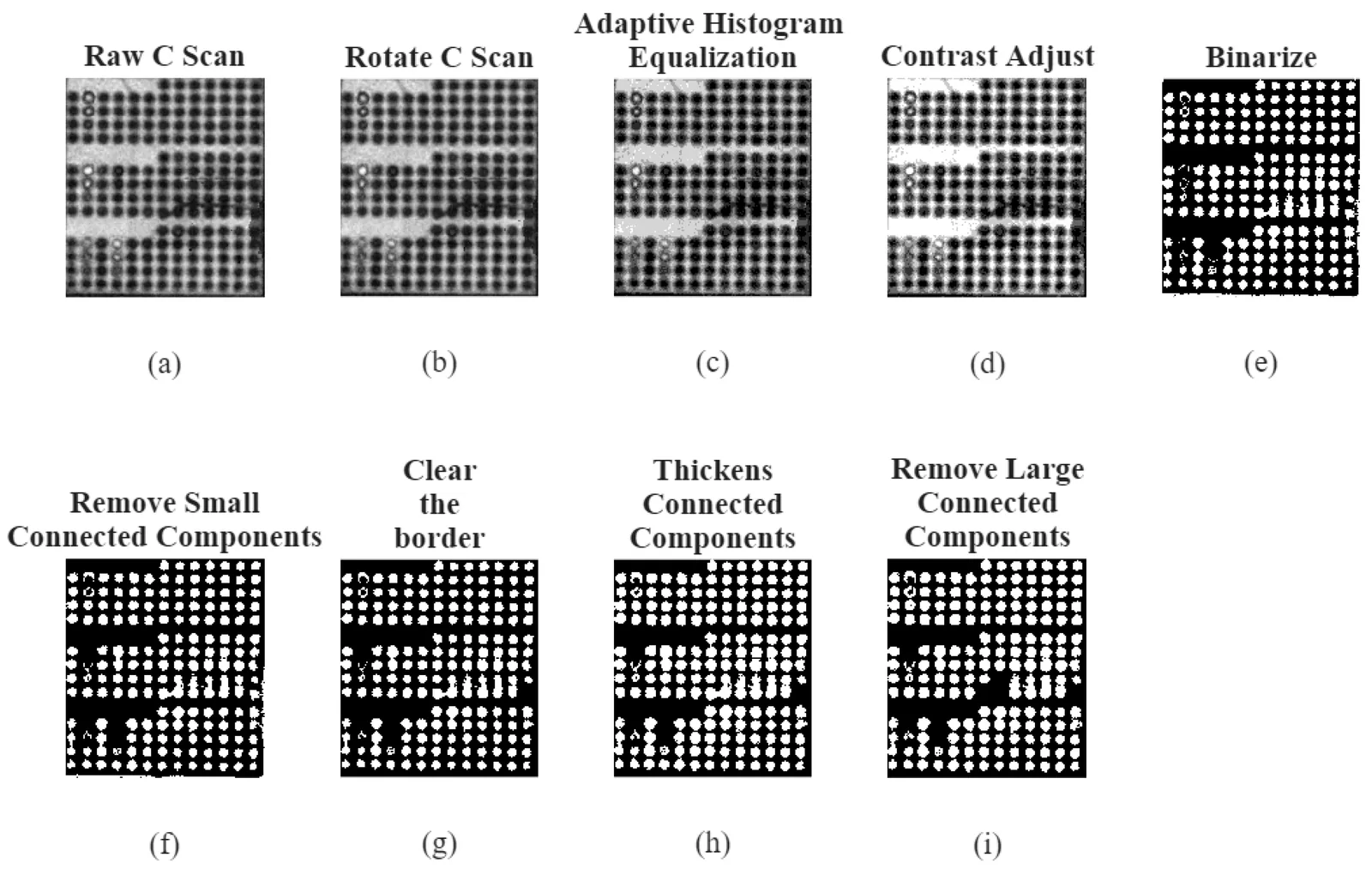
Thick sample image processing steps for feature detection.

The results from the data processing visualization for the thick base sample are shown in Figure 8. In the thin extrudate section, four features were paired together, and one of the hexagons was incorrectly labeled as a defect and thus removed from comparison. The average long diagonal error for the thin extrudate region, $e_{l_{d1}}$, after removing the paired features is −0.7 mm. Looking at the medium extrudate region, five hexagons were not detected, and several hexagons were combined in pairs due to what is another crack in the sample that occurs between the rows of features. This crack, unlike the thin sample, does not make the detected feature connected components greater than the λ threshold, so they remain in the detection and quantification. The average error, $e_{l_{d2}}$, in quantifying the long diagonal width for the medium extrudate region was −0.6 mm. Finally, for the thick extrudate region, all hexagons were correctly identified, resulting in an average long diagonal width error, $e_{l_{d3}}$, of −0.7 mm. Across the entire sample, the average error, $e_{l_{d}}$, is −0.7 mm.

Across all three samples, all hexagons were undersized relative to the optical microscopy measurements. This is likely due to the sharp corners of the hexagonal features causing the sound waves to scatter away from the transducer, resulting in a rounded appearance for the features.

Table 2 shows average long diagonal and hexagon height measurements, long diagonal and hexagon height standard deviation, long diagonal and hexagon height coefficient of variation, and percentage of features detected for the ultrasound algorithm. It also shows the average error between the microscope long diagonal and hexagon height and the ultrasound quantification measurement.

In terms of absolute long diagonal measurements, the clusters with the highest coefficient of variation (COV) for each of the thin, medium, and thick samples have a COV of 11.1%, 11.8%, and 9.4%, respectively. In terms of mm, these values relate to a standard deviation of 0.4 mm, 0.4 mm, and 0.3 mm for the thin, medium, and thick samples, respectively. In terms of an absolute measurement, this is quite a small standard deviation, especially since the scan and index resolutions are 0.2 mm, meaning the standard deviation is less than two indices in either the scan or the index dimensions.

The error between the microscopy and the ultrasound hexagon height measurements was determined similarly to that of Equation (12) as

(13) $$e_{h}\equiv h_{UT}-h_{actual}$$

where $e_{h}$ is the error between the actual hexagon height of each hexagon and the ultrasound hexagon height measurement.

**Figure 9:**
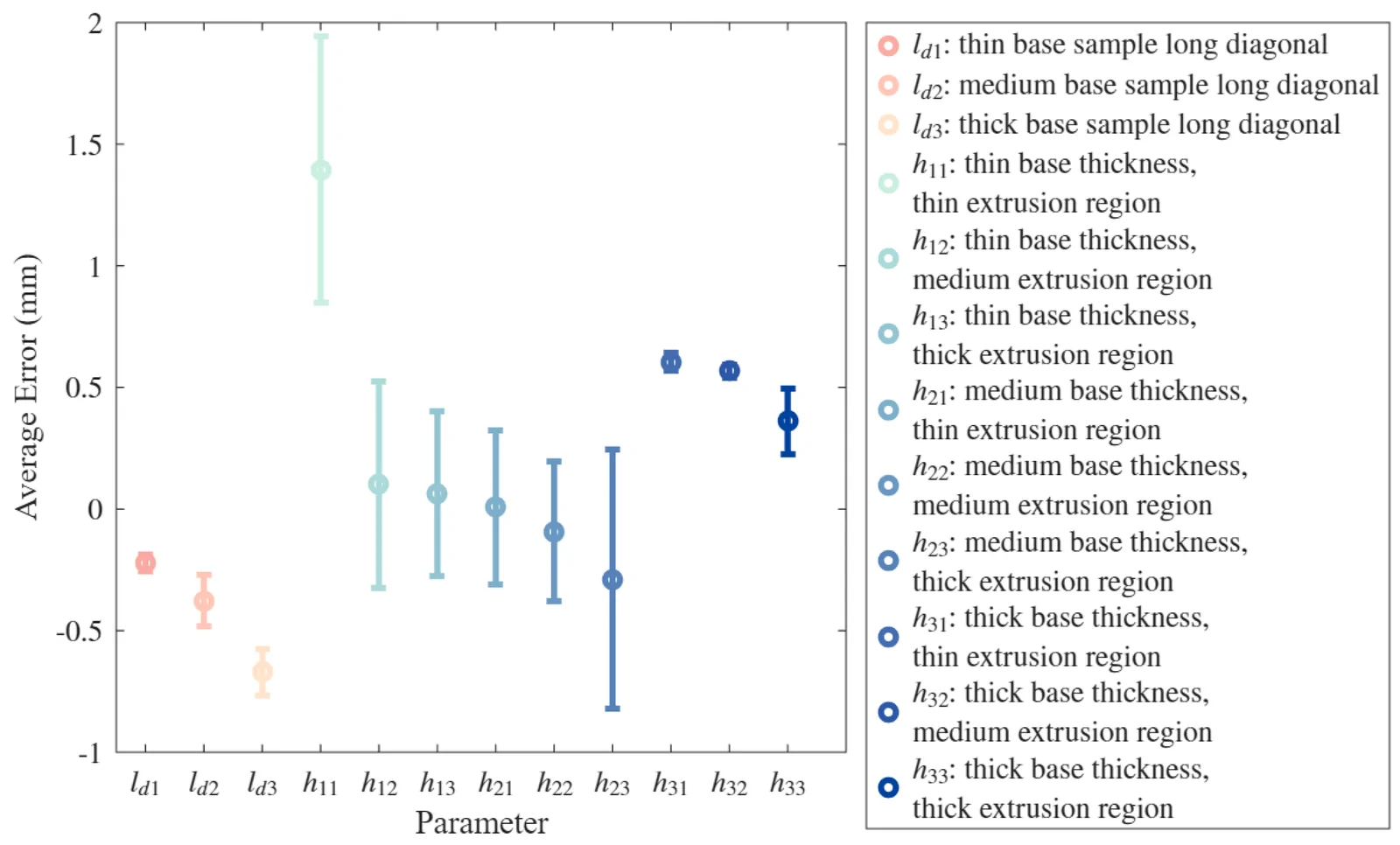
Error for long diagonal width and hexagon height with error standard deviation.

For the thin sample, the thin extrudate section has an average error, $e_{h}$, of 1.24 mm; however, the standard deviation for the error, as seen in Figure 9, is 0.60 mm. The average error for the medium extrudate region of the thin sample is 0.09 mm with a standard deviation of 0.42 mm. The thick extrudate region of the thin sample has an average hexagon height error of 0.06 mm and a standard deviation of 0.36 mm. The average error for the thin section is significantly higher than that of the medium and thick sections, likely due to how the last value greater than the back wall threshold value is selected. If a higher μ value is selected, this error will likely decrease; however, as discussed in Section 3, when selecting the μ parameter, it is not possible to increase μ across all specimens because of the thick base sample.

The medium and thick base thickness samples had relatively low average hexagon height errors, but a high variance as seen by the standard deviation in the error measurements. For the medium sample, the thin extrudate region has an average error of 0.02 mm and a standard deviation of 0.30 mm; the medium extrudate region has an average error of −0.09 mm and a standard deviation of 0.28 mm; and the thick extrudate region has an average error of −0.30 mm and a standard deviation of 0.56 mm. The thin extrudate region has an average error of 0.61 mm and a standard deviation of 0.04 mm; the medium extrudate region has an average error of 0.57 mm and a standard deviation of 0.03 mm; and the thick extrudate region has an average error of 0.36 mm and a standard deviation of 0.13 mm.

Figure 9 shows the measurement error between the ultrasound measurements and the Keyence measurements on the y-axis, with each different error parameter on the x-axis. On the x-axis, $l_{di}$ denotes the long diagonal width for the $i^{th}$ base thickness, as seen in Figure 1 and Table 1, and $h_{ij}$ denotes the hexagon height for the hexagon where *i* denotes the base thickness and *j* denotes the hexagon height, as seen in Figure 1 and Table 1.

When looking at the long diagonal measurements, the magnitude of the error is larger as a function of base thickness. This is likely due to greater attenuation dampening the sound energy more in the thicker samples, as the sound has to travel through more material before reflecting back to the transducer; more energy is lost in the process, and there is more internal scatter to disrupt the acoustic wave. Similarly, more beam spreading occurs as the base thickness increases by the time the sound wave reaches the base, so the wave is more out of focus, lowering the returned sound energy back to the transducer. The lower amount of sound energy returned to the transducer in the thicker samples results in a larger magnitude error for $l_{d}$ because the edges from the hexagons are most impacted by the lowered sound energy and increased beam spread, resulting in decreased precision in $l_{d}$.

Figure 9 also shows the error for the ultrasound height measurement compared to the optical microscopy measurements as a function of base thickness for each cluster of features. From this, the hexagon height measurements are not as precise as the long diagonal measurements using this method for all base thicknesses and hexagon extrusion heights. This is because, although the average error may seem low for hexagon thicknesses, the standard deviation in the error values is higher than that of the long diagonal measurements for both the thin base sample and the medium base sample, showing high variability in the method. Thus, more work is needed for this aspect of the quantification method. The height error for the thin base sample in the thin cluster is significantly higher than the other regions of the other samples. This is likely due to the large difference between the hexagon back wall intensity and the back wall threshold value, and the back wall detection being based on the last value greater than the back wall threshold. Thus, this is likely a lower limit on overall thickness (base thickness plus hexagon height) for this specific method due to the presence of significant internal echoes in the thin component.

When looking at the average error as a function of hexagon height, there is no trend between average error for either long diagonal or hexagon height as a function of hexagon extrusion height. This is because the average of the average long diagonal error for the thin, medium, and thick extrudate regions is −0.4 mm, −0.4 mm, and −0.5 mm, respectively. Since there is no meaningful increase or decrease as the hexagon height increases, there is no trend between hexagon height and long diagonal width average error. Then, for hexagon height, there is a trend because the average of the average hexagon thickness error for each extrudate region is 0.67 mm, 0.19 mm, and 0.04 mm for the thin, medium, and thick extrudate regions, respectively. This shows a decrease in error as the hexagon height increases. This makes sense because the back wall threshold is closer in value to the back wall threshold of the thickest base sample back wall reflection, as it has the lowest reflection intensity of the samples.

There appears to be no trend between detection rate and base thickness or extrusion height. This is because as the base thickness increases, the percentage of features detected does not increase or decrease. Similarly, when grouping the percentage of features detected based on extrusion height and averaging them, the percentage of features detected is 92.90%, 89.36%, and 98.58% for the thin hexagon height, medium hexagon height, and thick hexagon height, respectively. The percentage of features detected does not clearly increase or decrease as extrusion height increases; in other words, there does not appear to be a relationship between the two variables.

## 4. Conclusions

This study used spherically focused ultrasound to inspect a series of three stainless steel samples with three different base thicknesses manufactured via fused filament fabrication additive manufacturing. These samples had hexagons with nominal long diagonals of 4.06 mm and were extruded to three heights with nominal heights of 1.02 mm, 1.52 mm, and 2.03 mm, to quantify a large quantity of features extruded on the opposing surface to simulate features extruding from an interior surface of a component. Such features were extruded from bases of various thicknesses and extruded to various thicknesses. An image processing algorithm was used to quantify both the long diagonal of these hexagonal features and the hexagon height of the features from the front surface of the sample. The image processing techniques used included adaptive histogram equalization, adaptive binarization, and thickening. These measurements were compared to optical microscope measurements to determine the difference between the actual measurement and the ultrasound measurement.

It was concluded that the hexagon long diagonal was always undersized relative to the optical microscope measurement by a consistent range of 0.1 to 0.7 mm, likely due to scattering at the sharp feature edges. Similarly, it was determined that the long diagonal error increases as a function of base thickness, likely due to increased attenuation from sound traveling through more material back to the transducer. No trends were observed between hexagon extrusion height and average long diagonal error; however, a trend was observed between hexagon height and average hexagon height error. Similarly, no trend is apparent between the detection rate and base thickness or detection rate and extrusion height. Lastly, when looking at the thickness measurements, this aspect is less precise compared to the long diagonal measurements, as the standard deviation of the error is significantly higher. Thus, more development needs to be performed in this area, and it is currently in development for a future manuscript.

In terms of limitations, one limitation to this method is that it assumes all features begin extruding at the same depth into the component. Since the features are all being measured at the base where the features begin extruding in this case, but for a sample with features occurring at different depths, a modification of the present approach would be required.

Future work should investigate the automation of the feature detection and quantifying them separately from the non-defective areas. Another aspect of future work should focus on thickness quantification, as that is presently the largest quantification error.

## Figures and Tables

**Table 1:** Test matrix for the samples manufactured showing the nominal designed values of the base thickness, the hexagon cluster height, and hexagon long diagonal.

Sample Base Name	Base Thickness ($b_{i},\;i=1,\;2,\;3$) (mm)	Hexagon Height ($h_{j},\;j=1,\;2,\;3$) (mm)	Hexagon Long Diagonal ($l_{d}$) (mm)
Thin Sample	1.27	1.02, 1.52, 2.03	4.06
Medium Sample	2.54	1.02, 1.52, 2.03	4.06
Thick Sample	4.83	1.02, 1.52, 2.03	4.06

**Table 2:** Long diagonal width and hexagon height measurements for the ultrasound image processing method for each sample compared to the Keyence microscope measurements.

			Average Value (mm)	Standard Deviation (mm)	Coef. of Variation (%)	Percentage of Features Detected	Average Meas. Error (mm)
Thin Sample	Thin Section	$l_{d1}$	3.8	0.2	5.3	87.23	−0.1
		$h_{11}$	2.38	0.60	19.36		1.24
	Medium Section	$l_{d2}$	3.6	0.4	11.1	87.23	−0.3
		$h_{12}$	1.81	0.37	20.44		0.09
	Thick Section	$l_{d3}$	3.6	0.3	8.3	95.74	−0.3
		$h_{13}$	2.09	0.34	15.3		0.07
	Total Sample	$l_{d}$	3.6	0.3	8.3	90.07	−0.3
		*h*	–	–	–		–
Medium Sample	Thin Section	$l_{d1}$	3.4	0.4	11.8	100	−0.5
		$h_{21}$	1.12	0.30	27.0		0.02
	Medium Section	$l_{d2}$	3.4	0.2	5.9	100	−0.3
		$h_{22}$	1.51	0.26	17.1		−0.09
	Thick Section	$l_{d3}$	3.4	0.2	5.9	100	−0.5
		$h_{23}$	1.71	0.57	31.3		−0.30
	Total Sample	$l_{d}$	3.4	0.3	8.8	100	−0.4
		*h*	–	–	–		–
Thick Sample	Thin Section	$l_{d1}$	3.2	0.3	9.4	91.49	−0.7
		$h_{31}$	1.69	0.05	3.0		0.61
	Medium Section	$l_{d2}$	3.3	0.2	6.1	80.85	−0.6
		$h_{32}$	2.12	0.04	1.9		0.57
	Thick Section	$l_{d3}$	3.2	0.2	6.3	100	−0.7
		$h_{33}$	2.52	0.14	5.6		0.36
	Total Sample	$l_{d}$	3.2	0.3	9.4	90.78	−0.7
		*h*	–	–	–		–

## Data Availability

The raw data supporting the conclusions of this article will be made available by the authors on request.
